# Quantification of Free Radicals from Vaping Electronic Cigarettes Containing Nicotine Salt Solutions with Different Organic Acid Types and Concentrations

**DOI:** 10.1021/acs.chemrestox.4c00065

**Published:** 2024-05-22

**Authors:** Lillian N. Tran, Guodong Rao, Nicholas E. Robertson, Haylee C. Hunsaker, Elizabeth Y. Chiu, Brett A. Poulin, Amy K. Madl, Kent E. Pinkerton, R. David Britt, Tran B. Nguyen

**Affiliations:** † Department of Environmental Toxicology, University of California, Davis, Davis, California 95616, United States; ‡ Department of Chemistry, University of California, Davis, Davis, California 95616, United States; § Center for Health and the Environment, University of California Davis, Davis, California 95616, United States

## Abstract

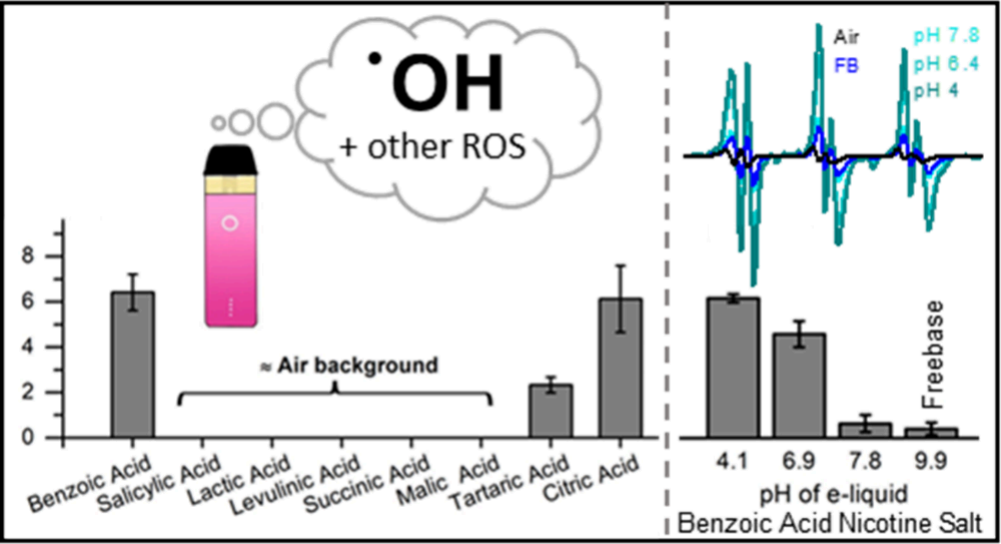

Electronic (e-) cigarette formulations containing nicotine salts from a range of organic acid conjugates and pH values have dominated the commercial market. The acids in the nicotine salt formulations may alter the redox environment in e-cigarettes, impacting free radical formation in e-cigarette aerosol. Here, the generation of aerosol mass and free radicals from a fourth-generation e-cigarette device was evaluated at 2 wt % nicotine salts (pH 7, 30:70 mixture propylene glycol to vegetable glycerin) across eight organic acids used in e-liquids: benzoic acid (BA), salicylic acid (SLA), lactic acid (LA), levulinic acid (LVA), succinic acid (SA), malic acid (MA), tartaric acid (TA), and citric acid (CA). Furthermore, 2 wt % BA nicotine salts were studied at the following nicotine to acid ratios: 1:2 (pH 4), 1:1 (pH 7), and 2:1 (pH 8), in comparison with freebase nicotine (pH 10). Radical yields were quantified by spin-trapping and electron paramagnetic resonance (EPR) spectroscopy. The EPR spectra of free radicals in the nicotine salt aerosol matched those generated from the Fenton reaction, which are primarily hydroxyl (OH) radicals and other reactive oxygen species (ROS). Although the aerosol mass formation was not significantly different for most of the tested nicotine salts and acid concentrations, notable ROS yields were observed only from BA, CA, and TA under the study conditions. The e-liquids with SLA, LA, LVA, SA, and MA produced less ROS than the 2 wt % freebase nicotine e-liquid, suggesting that organic acids may play dual roles in the production and scavenging of ROS. For BA nicotine salts, it was found that the ROS yield increased with a higher acid concentration (or a lower nicotine to acid ratio). The observation that BA nicotine salts produce the highest ROS yield in aerosol generated from a fourth-generation vape device, which increases with acid concentration, has important implications for ROS-mediated health outcomes that may be relevant to consumers, manufacturers, and regulatory agencies.

## 1 Introduction

After the American electronic (e-) cigarette company JUUL introduced nicotine salts to the market in 2015 to be used with their fourth generation pod devices, other e-cigarette brands also quickly adopted the production of nicotine salts.^1^ E-liquid solutions containing nicotine salts are now considered the most popular e-liquids to vape with pods and disposable e-cigarette devices.^1−6^ Unlike e-liquids using freebase nicotine, nicotine salt solutions contain the addition of an organic acid or a mixture of organic acids to form an ionic pair, or a salt, with nicotine. Nicotine salt e-liquid solutions are often available with high concentrations of nicotine (up to 5% or 50 mg/mL) in a mixture of propylene glycol (PG) and vegetable glycerin (VG).^7^ PG and VG are hygroscopic, and thus, water is also one of the most abundant components of e-liquids, present at up to 18 wt % in previously studied vape products.^8−10^ The pH of commercial nicotine salt e-liquids has been measured to be as low as 3.6,^11^ depending on the exact formulation of nicotine salt and ratio of the organic acid to nicotine. Note that the term “pH” in this work and others should be used for relative comparisons between e-liquids only. Although pH measurements of PG/VG-based e-liquids with a potentiometric probe are stable upon addition of water and provide a good approximation of the acid content in solution,^12^ the values obtained may not be an accurate quantification of hydronium ion activity due to the lower water environment in e-liquids, even upon dilution.^12,13^ Freebase nicotine e-liquids are generally available at a 3–20 mg/mL nicotine concentration. The freebase nicotine e-liquids have a measured pH of generally 9–10 in commercial formulations, but such alkaline nicotine formulations can cause a bitter and harsh sensation in the throat when inhaling the aerosol, which have been reported as unappealing to users.^14−16^ The role of the organic acid is to allow users to vape at higher concentrations of nicotine without compromising taste as well as increasing nicotine delivery deeper into the lungs, resulting in higher overall user satisfaction.^7^ Vape users indicate that vaping nicotine salts gave better sensory experiences in terms of smoothness and taste compared to freebase nicotine.^15,16^

The JUUL patent for nicotine salts mentions 32 different organic acid formulations,^7^ and a recent study found six different organic acids in 23 commercial e-liquids,^11^ including popular choices such as benzoic acid, lactic acid, and levulinic acid. Despite the current popularity and diversity of nicotine salt e-liquids, there are few studies on the chemical and toxicological properties of e-cigarette aerosol following the addition of high concentrations of corrosive organic acids to the e-liquid. There are numerous potential chemical and toxicological effects from the addition of conjugate organic acids to nicotine that merit additional investigation. First, metal solubility is generally higher at lower solution pH,^17^ representing an additional health concern of e-cigarette use. It is known that e-cigarette devices may leach trace metals into the e-liquid and aerosol.^18−23^ This metal leaching may be facilitated by both the water and organics in the e-liquid as polyols and organic acids can extract metal ions.^24^ Thus, it may be hypothesized that nicotine salt e-liquids can increase the leaching of metals from coil surfaces when compared to freebase nicotine e-liquids and that leaching of metals may increase as the organic acid concentration increases. These metals may be directly toxic as well as participate in redox chemistry in the e-cigarette vessel.

Second, organic acids may promote redox chemistry when interacting with trace metals in the e-liquid solution and aerosol, including the Fenton redox reaction that produces oxygenated radical species, i.e., reactive oxygen species (ROS), such as highly reactive hydroxyl (OH) radicals. The classical Fenton reaction involves the cycling between ferrous (Fe(II)) and ferric (Fe(III)) ions in water, while reducing hydrogen peroxide to OH, superoxide, and other ROS. Transition metals such as iron and copper will complex with organic acids.^25^ Some organic acids such as citric acid and oxalic acid were found to accelerate radical production, whereas organic acids like malonic acid were found to suppress radical production.^26^ Thus, the different organic acid formulations available on the market^7^ may either promote or suppress redox chemistry in e-cigarettes. However, it is not yet clear whether the chemistry of these organic acids tested in other applications can be extrapolated to a realistic vaping condition.

The increased concentrations of both redox-active metals and certain organic acid ligands may be hypothesized to promote the production of ROS in the e-cigarette aerosol, which has direct implications for cytotoxicity, oxidative stress, and other adverse health outcomes when inhaled. Although previous studies have measured free radicals from e-cigarette devices using solvent only or freebase nicotine in solvent,^27−32^ there is yet to be such a study for nicotine salt systems. Previous studies also measured radical formation from added flavorants, without emergence of a discernible pattern.^28,33^ This paper investigates (1) whether the free radical species produced from vaping nicotine salt e-liquids can be identified as ROS that are comparable to those produced by Fenton-like chemistry, (2) which organic acids in nicotine salt formulations suppress or promote radical formation in the inhalable aerosol from a fourth-generation e-cigarette pod device with a realistic vaping regimen, and (3) whether increasing acid concentrations contribute to higher radical formation in the e-cigarette aerosol. Results from this paper have implications for the regulation, production, and use of nicotine salts in a manner that reduces harm.

## 2 Methods

### 2.1 E-Liquid Formulations

**Figure 1:**
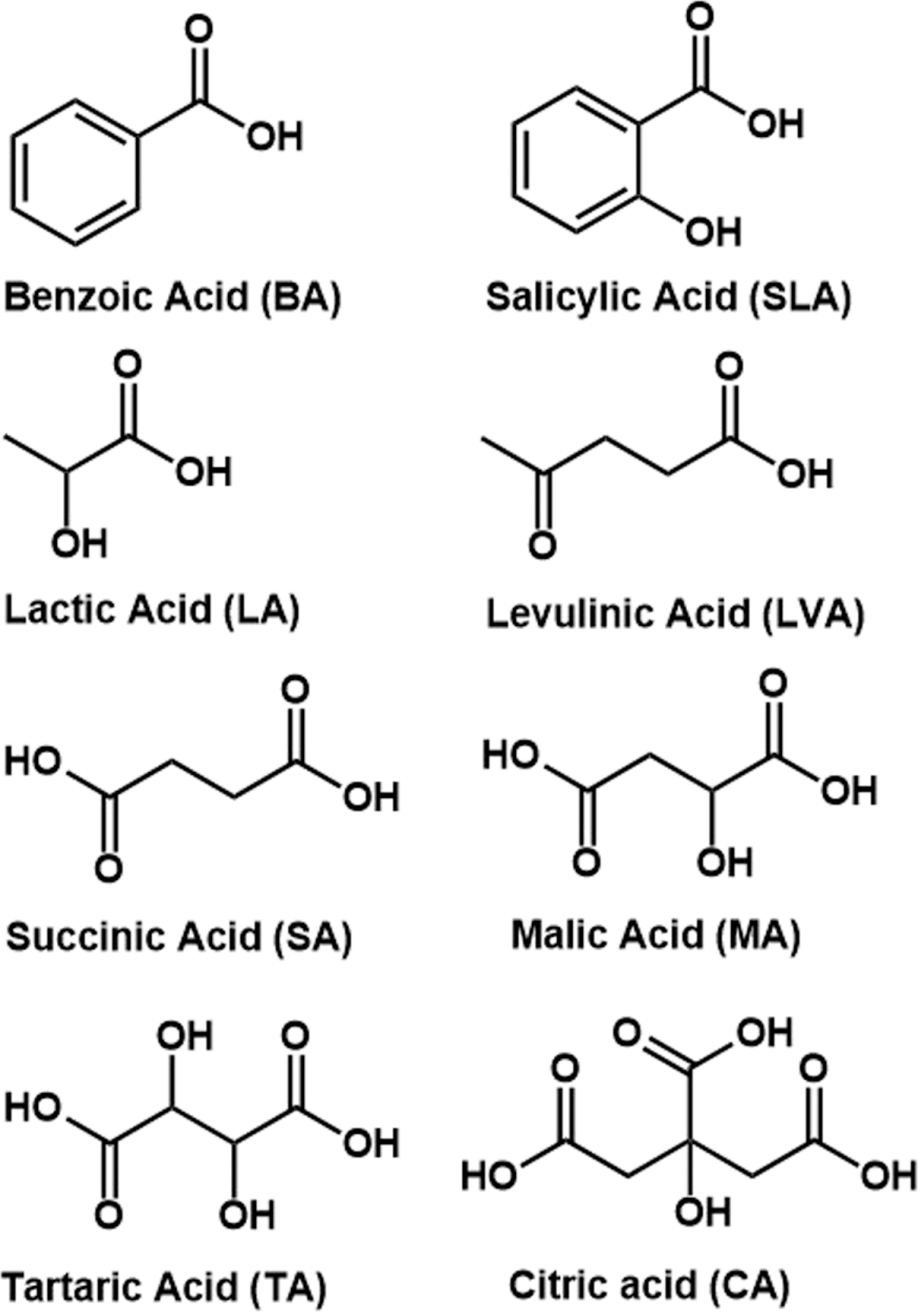
Chemical structures, common names, and abbreviations for the eight organic acids tested in this study in nicotine salt formulations.

All e-liquid formulations were prepared with 2% (w/w) nicotine in 30% PG and 70% VG (purities 99%, 99%, and ≥99.5%, respectively, from Sigma-Aldrich). Freebase nicotine (2%, FB) e-liquids were made by dissolving nicotine in PG and VG without the addition of organic acid. Following the JUUL patent,^7^ 2% (w/w) nicotine salts were made by mixing together nicotine, organic acid, PG, and VG with heating at 40 °C to avoid chemical degradation and stirred for up to 2 h until full dissolution. Eight organic acids were chosen for study (Figure 1) based on commercial market relevance: benzoic acid (BA), lactic acid (LA), levulinic acid (LVA), succinic acid (SA), salicylic acid (SLA), tartaric acid (TA), malic acid (MA), and citric acid (CA). Of these, benzoic acid and lactic acid are the most commonly used in commercial products at present.^7,11,34^ The unadjusted pH values for all e-liquid formulations in this study are shown in Table 1. For studies on radical formation from nicotine salts with different types of organic acid conjugates, e-liquids were formulated at equimolar ratios (1:1) of nicotine to acid and adjusted to neutral pH using sodium hydroxide (NaOH) when required. BA, TA, and CA (≥99.5% purity) were purchased from Sigma-Aldrich. LA (90% purity) was purchased from Acros Chemicals, and MA (>99% purity) was manufactured by Indofine Chemical Company but purchased from Thomas Scientific. For studies on radical formation from the different acid concentration, nicotine salts at three different molar ratios of nicotine to benzoic acid were tested: 1:2 (pH ∼ 4), 1:1 (pH ∼ 7), and 2:1 (pH ∼ 8).

**Table 1:** Compositions of the E-Liquid Solutions Studied in This Work and Their Unadjusted pH Values at the Specified Nicotine to Acid Ratio

**e-liquid solution**	**pH**
2% freebase	9.9
2% nicotine salt (BA 2:1)	7.8
2% nicotine salt (BA 1:1)	6.8
2% nicotine salt (BA 1:2)	4.0
2% nicotine salt (SLA 1:1)	4.6
2% nicotine salt (LA 1:1)	7.3
2% nicotine salt (LVA 1:1)	5.8
2% nicotine salt (SA 1:1)	4.8
2% nicotine salt (MA 1:1)	4.2
2% nicotine salt (TA 1:1)	3.7
2% nicotine salt (CA 1:1)	3.7

### 2.2 pH Measurement

pH measurements of e-liquids followed the protocols reported in previous studies.^11,32,34,35^ The acidity and basicity of e-cigarette solutions have also been measured with other techniques such as proton NMR^36^ and X-ray spectroscopy.^37^ The pH values discussed here do not necessarily represent absolute hydronium activity in solution.^13^ Instead, they are discussed within a relative context in this work and are provided to extrapolate our results to commercial e-liquids with proprietary formulations for which pH values have been measured using similar methods. E-liquids were diluted in water by 90% (by volume), thoroughly mixed, and measured with a pH meter (FP20,Mettler-Toledo) calibrated with commercial buffer solutions. All pH measurements were measured in triplicate. Bourgart et al. found that a dilution factor from 2 to 200 yielded similar pH measurements.^12^ Note that the pH measurement by Harvanko et al. of commercial nicotine salts matched the pH measurements in all the formulated nicotine salts in this study, except for LA. Several nicotine LA salts were reported by Harvanko et al. to have a pH of ∼4, whereas we measured the pH of a 1:1 nicotine to LA nicotine salt to be around pH 7. It is possible that the higher (5%) nicotine salt concentration greatly reduces the pH or the molar ratio of those commercial nicotine salts measured by Harvanko et al. was not 1:1.

### 2.3 Generation of Aerosol and Sampling of Free Radicals

The generation of vaping aerosol follows a protocol previously reported by our group.^38^ Briefly, a refillable fourth-generation Vaporesso XROS 2 pod device (Shenzhen Smoore Technology Limited) with a battery capacity of 1000 mAh and the XROS 1.2 Ω pod was used for aerosol generation. An average vacuum flow of 2.18 ± 0.09 L/min was used for device activation, resulting in an average puff volume of 72.8 ± 3.1 mL with a 2 s puff duration and 2 puff per minute interval. The puffing regimen was controlled by solenoid valves that were operated with a time relay controller (PTR4-SP, Changzhou Xuchuang Info. Tech. Co.). Each set of samples was collected with a new pod to minimize cross-contamination and coil aging effects.^32,38−40^

**Figure 2:**
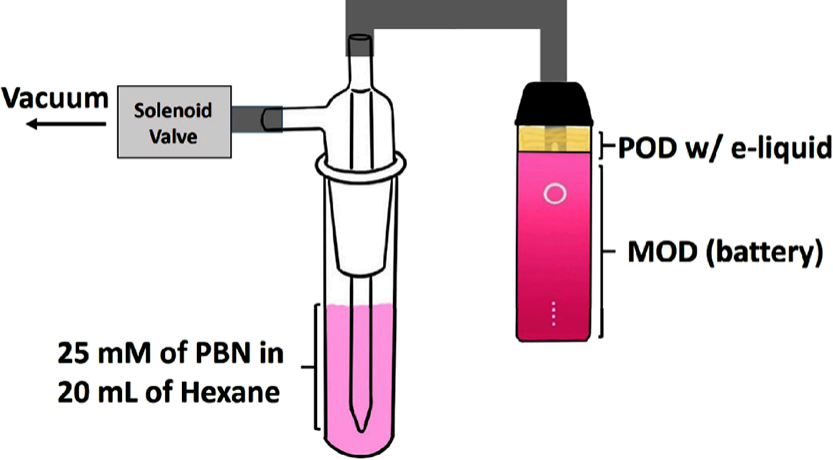
Fourth generation electronic cigarette vaping and aerosol sampling setup.

Free radicals were spin-trapped for spectroscopic analysis with a chemical reagent. Adapted from previously reported methods with e-cigarette aerosol, the spin trapping procedure was optimized in this study using 25 mM *N*-*tert*-butyl-α-phenylnitrone (PBN, >98% purity, Cayman Chemicals) in 20 mL of hexane.^27,28,30,31^ The Vaporesso XROS pod device was vaped through an impinger filled with spin trap solution (Figure 2). After 70 puffs, the sample solution was evaporated using a rotary evaporator at 35 °C and reconstituted in 400 μL of toluene to dissolve all solids. A total of 300 μL of the solution was directly transferred to a quartz X-band EPR tube and subsequently degassed by sparging with ultrahigh purity nitrogen (N_2_). In our optimization tests, it was found that N_2_ sparging yielded more consistent results and sharper peaks compared to those of the freeze–pump–thaw method using a Schlenk Line. Solvent evaporation due to N_2_ sparging was minimal and was accounted for by precise measurements of the solvent line in the EPR tube. All samples were collected and analyzed in triplicate.

To measure the aerosol mass (mg per puff) generated by the e-cigarette device, the pod was weighed before and after each sample and the mass difference was divided by the number of puffs.^41^ The mass difference of each collection (mass of e-liquid consumed) was also equated to the mass of aerosol formed, which was used to normalize the concentration of radicals detected in each sample.

Spin-trapped radicals from the Fenton reaction were generated by mixing a 1 mL aqueous solution of ferrous sulfate heptahydrate (FeSO_4_·7H_2_O, Sigma-Aldrich, >99% purity) and H_2_O_2_ (Sigma-Aldrich, 50 wt % in water) at final concentrations of 200 and 100 μM, respectively. Immediately after combining Fe(II) and H_2_O_2_, 300 μL of 10 mM PBN in toluene was added, and the mixture was agitated for >1 min. The toluene layer was extracted, evaporated, and reconstituted in 300 μL of toluene for N_2_ sparging following the same procedure used for the vape samples.

### 2.4 Analysis of Free Radicals Using EPR Spectroscopy

Electron paramagnetic resonance (EPR) spectroscopy was performed at the CalEPR center in the Department of Chemistry at the University of California, Davis. X-band (9.4 GHz) continuous wave EPR spectra were recorded on a Bruker Biospin EleXsys E500 spectrometer with a super high Q resonator (ER4122SHQE) at room temperature. All spectra were recorded under slow-passage, nonsaturating conditions. Spectrometer settings were as follows: microwave frequency = 9.37 GHz; microwave power = 0.63 mW; conversion time = 120 ms; modulation frequency = 100 kHz; modulation amplitude = 0.5 gauss.

To quantify the PBN-radical adducts, 2,2,6,6-tetramethylpiperidine 1-oxyl (TEMPO) at 0.5, 2, 4, 20, and 40 μM in toluene were used as external standards.^27,28,30,31^ The double integrals of spin-adduct EPR signals, normalized by the quality (*Q*) factor of the resonator, were used for quantification.^42^ In addition, all samples were normalized by the grams of aerosol formed to correct for any uncertainty in the vaporization and directly compare across all e-liquids. The background concentrations of radicals were measured by sampling lab air through the spin trap solution for 70 puffs (“air background”). All data presented in nanomoles of radicals per gram of aerosol formed have been air background-corrected.

## 3 Results and Discussion

### 3.1 Identity of Radicals in E-Cigarette Aerosol

**Figure 3:**
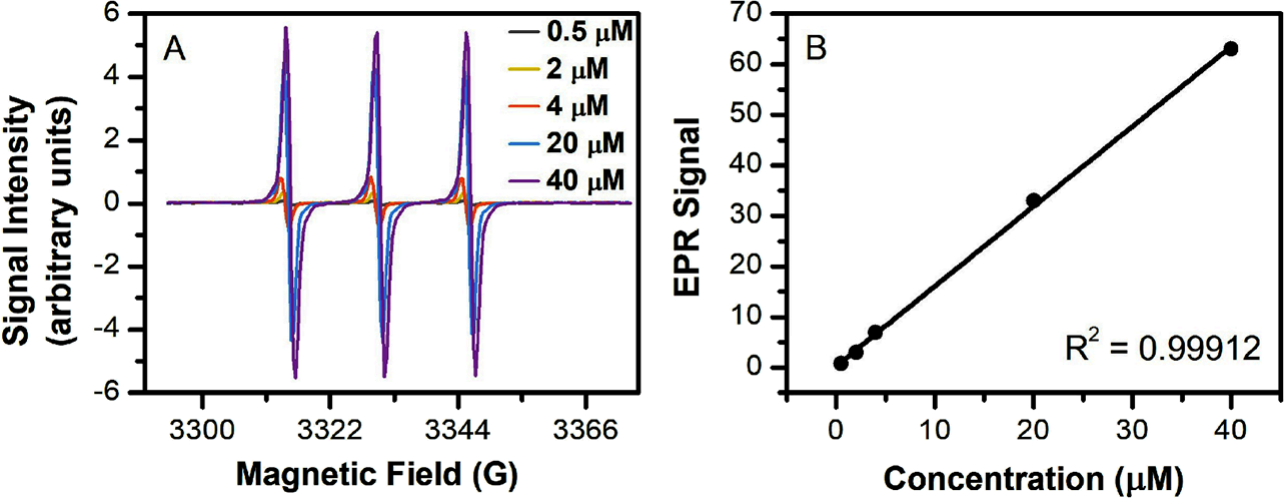
EPR signal calibration with TEMPO standard solutions in toluene.

**Figure 4:**
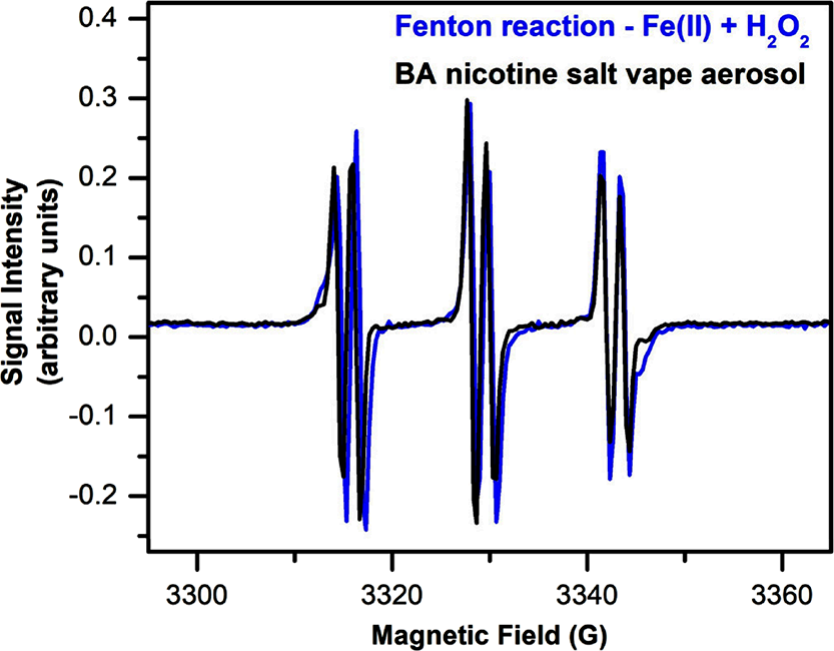
EPR spectra of the PBN–OH adduct from Fe(II) + H_2_O_2_ versus the spectra of the PBN–radical adduct from a BA nicotine salt aerosol vape sample.

EPR signal calibrations with TEMPO are shown in Figure 3. The *Q* factor for the TEMPO standards was 6000 but was reduced to 1000–1200 for all aerosol samples, likely due to the polar solvents present in the e-liquids; this was corrected during the radical quantification. To provide insights into the identities of the radicals produced in the vaping process tested here, we compared the EPR spectra of an aerosol sample produced by the 1:1 BA nicotine salt with those of the classical Fe(II)/H_2_O_2_ dark Fenton reaction that is often used as a standard for OH radicals. While sample processing may affect the line shape, the PBN spin-trapped EPR signals of both samples show nearly identical hyperfine splitting patterns, with *a*^1^H = 5.3 MHz, or 1.89 G, and *a*^14^N = 38.3 MHz, or 13.7 G (Figure 4). These hyperfine coupling constants are usually used to distinguish different trapped radical species. It thus suggests that the radicals produced from vaping BA nicotine salts are most likely identical with the ROS produced by dark Fenton, although the data do not insinuate that the radicals in the vape sample are produced by Fenton chemistry. These results via EPR are consistent with the detection of OH radicals in freebase nicotine vape aerosol through product analysis.^33^ Other solution-phase ROS are generally present in the chemical equilibria concurrently with OH (e.g., O_2_^·–^, HO_2_, and H_2_O_2_), although they are less reactive.

### 3.2 Different Organic Acid Nicotine Salts at pH 7 and 1:1 Molar Ratio

**Figure 5:**
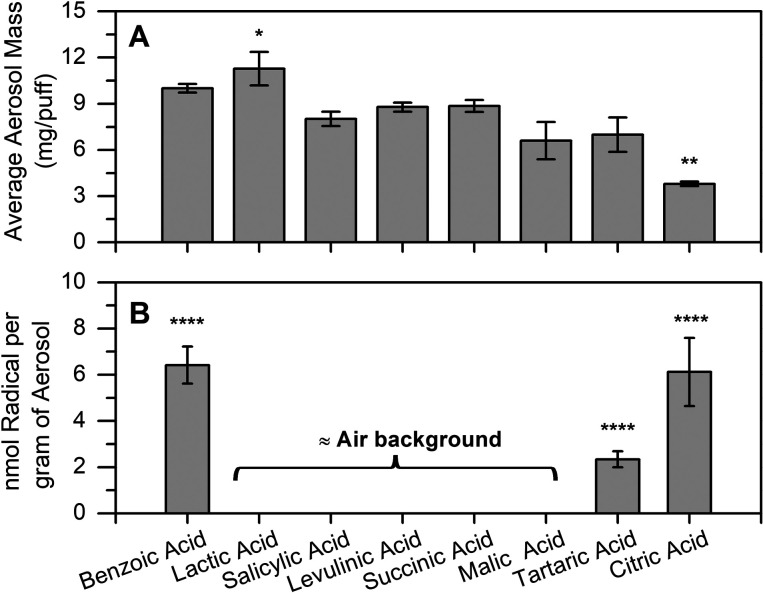
(A) Aerosol mass (mg per puff) and (B) radicals (nmol per gram of aerosol formed) from vaping nicotine salts with different organic acids at pH 7. Data have been corrected for air background. Asterisks denote statistically significant differences using one-way ANOVA: *p < 0.1; **p < 0.05; ****p < 0.001. Triplicate data in A were compared against the mean ± SD, and triplicate data in B were compared against the air background.

The aerosol mass produced by the device did not significantly vary across the different nicotine salt types at 1:1 molar ratio and neutral pH (Figure 5A), except that the aerosol yields from LA were slightly higher than the mean (8.0 ± 2.3 mg/puff) with a *p*-value of 0.09 and those from CA were significantly lower than the mean with a *p*-value of 0.03 using one-way ANOVA. These data are tabulated in Table 2. It is possible that the neutralization ratio of nicotine/acid affects the atomization efficiency. The two most efficiently aerosolized e-liquids at the 1:1 molar ratio were the salts from the monoacids BA and LA. Diacid salts such as SLA, LA, SA, MA, and TA produced less aerosol mass per puff than the monoacids, and the triacid CA produced the lowest amount of aerosol. CA was found to form 2:1 neutralization ratios with nicotine, similar to TA and MA in an earlier study;^43^ thus, it is not clear why its aerosol formation is approximately half those of TA and MA if nicotine ratios were the only factor responsible. It is possible that the vaping environment requires more CA to fully neutralize nicotine. It should be noted that commercial CA e-liquids may have different proportions than the 1:1 molar ratio used in this study for radical formation, although the exact ratios are difficult to discern as commercial e-liquid formulations are often proprietary. However, these data are consistent with a report by JUUL Laboratories that CA nicotine salt e-liquids delivered the lowest amount of nicotine of the 11 varieties they tested,^44^ if it can be assumed that nicotine delivery correlates with aerosol mass.

**Table 2:** Aerosol Mass (mg per Puff) and Radicals (nmol and nmol per Gram of Aerosol Formed) (Mean ± SD) from Vaping Nicotine Salts with Different Organic Acids^a^

organic acid	aerosol mass (mg/puff)	total radicals detected (nmol)	nmol radicals per g aerosol
benzoic acid	10.00 ± 0.27	4.48 ± 0.45	6.42 ± 0.80
lactic acid	11.28 ± 1.09	LOB	LOB
salicylic acid	8.02 ± 0.47	LOB	LOB
levulinic acid	8.78 ± 0.30	LOB	LOB
succinic acid	8.86 ± 0.39	LOB	LOB
malic acid	6.61 ± 1.21	LOB	LOB
tartaric acid	6.99 ± 1.12	1.06 ± 0.29	2.34 ± 0.34
citric acid	3.80 ± 0.15	1.63 ± 0.41	6.12 ± 1.47
freebase	10.00 ± 0.55	0.36 ± 0.23	0.56 ± 0.37

a LOB (limit of blank) indicates a value statistically indistinguishable from the air background value.

**Figure 6:**
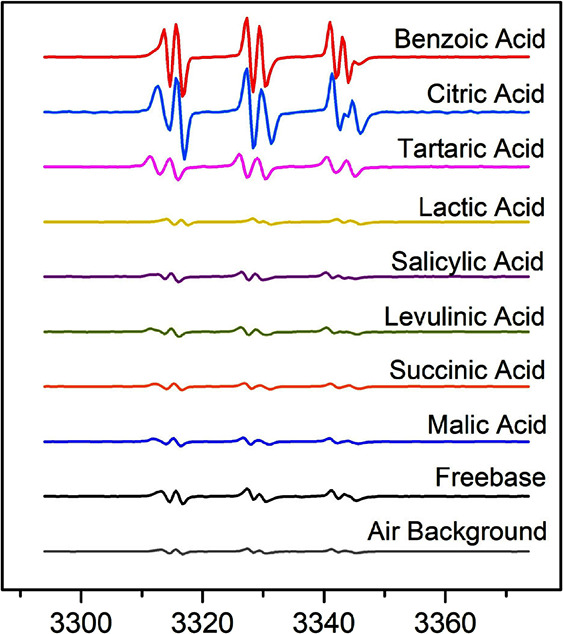
EPR spectra of PBN–radical adducts detected in the e-cigarette aerosol from vaping nicotine salts with different organic acids in a 1:1 ratio with nicotine at pH 7. Nicotine salt data are compared against freebase nicotine vape aerosol and air background controls. Data are normalized using the aerosol mass generated.

Free radicals were detected in significant quantities only in the benzoic acid (BA), tartaric acid (TA), and citric acid (CA) vape samples, whereas the signals from vape samples with other nicotine salts were indistinguishable within uncertainty to the air background (Table 2, Figure 5B); the corresponding EPR spectra are shown in Figure 6. Among the different nicotine salts, the BA and CA nicotine salts were observed to have the most significant amounts of radicals per gram of aerosol formed (Figures 5B and 6). The absolute quantity of radicals detected for the CA nicotine salt in 70 puffs was lower than the BA nicotine salt, but since the aerosol mass produced by the citrate salt was significantly less, it amounted to a similar average nanomoles of radical per gram of aerosol formed compared to the benzoate salt. Radicals were also detected with the TA nicotine salt but were less abundant than the BA and CA nicotine salts. While CA and TA have been shown to be ligands for iron (either Fe(II) or Fe(III) species) in Fenton-like reactions in water^25,26,45^ and the generation of ROS from these salts may be expected, there is no such literature showing BA to be a participant in Fenton-like chemistry. Thus, a surprising result was that BA salts yielded the highest concentration of radicals per gram of aerosol formed. These results have implications for the commercial market as e-liquids with BA salts are one of the most popular choices available for consumers.

While radical formation is an important part of the story, these data also suggest that radical scavenging needs to be considered. Of the aromatic acids, BA produced the highest ROS yield, while SLA did not produce ROS at a level above background air, despite SLA being structurally analogous to BA except for the addition of one ortho hydroxy group. Consistent with our spin-trapped ROS measurement results, a recent *in vitro* study also found that BA nicotine salt vaping aerosol from JUUL devices was the only tested formulation that produced significant oxidative stress response, whereas SLA nicotine salts, freebase nicotine salts, and the organic acids themselves did not.^46^ BA has not been studied for radical production but has been studied for its radical scavenging abilities.^47,48^ BA has relatively low scavenging capabilities;^47^ whereas benzoic acids with additional methoxy and hydroxy groups have significantly higher radical scavenging capabilities.^47,48^ This is consistent with the SLA and BA results in our work. Thus, even if SLA can interact with metals to produce ROS, it is likely that it scavenges more radicals than it produces, resulting in a negligible net effect.

Of the aliphatic acids, tartaric acid (TA) is analogous to malic acid (MA) except with one additional hydroxy group, and yet, we observed detectable ROS from TA nicotine salt e-liquids but not MA. Lactic acid (LA) is similar to MA except with one fewer carboxylic group. A study on the free radical scavenging capacity of kefir found that goat kefir, which had higher amounts of LA and MA compared to cow kefir, had higher free radical scavenging capacity.^49^ Their results are consistent with this work (Figures 5 and 6), although the matrix and environments are different. It should also be noted that aerosol produced from vaping the nicotine salts containing SA, LA, LVA, SA, and MA had levels of radicals even lower than the aerosols produced from vaping freebase nicotine (Table 2, Figure 6), suggesting that the addition of these acids may lower radical levels in the aerosol through scavenging. These data, taken together, suggest that observable ROS may result from a balance between radical scavenging and radical formation.

### 3.3 Different Molar Ratios of Benzoic Acid Nicotine Salts

**Figure 7:**
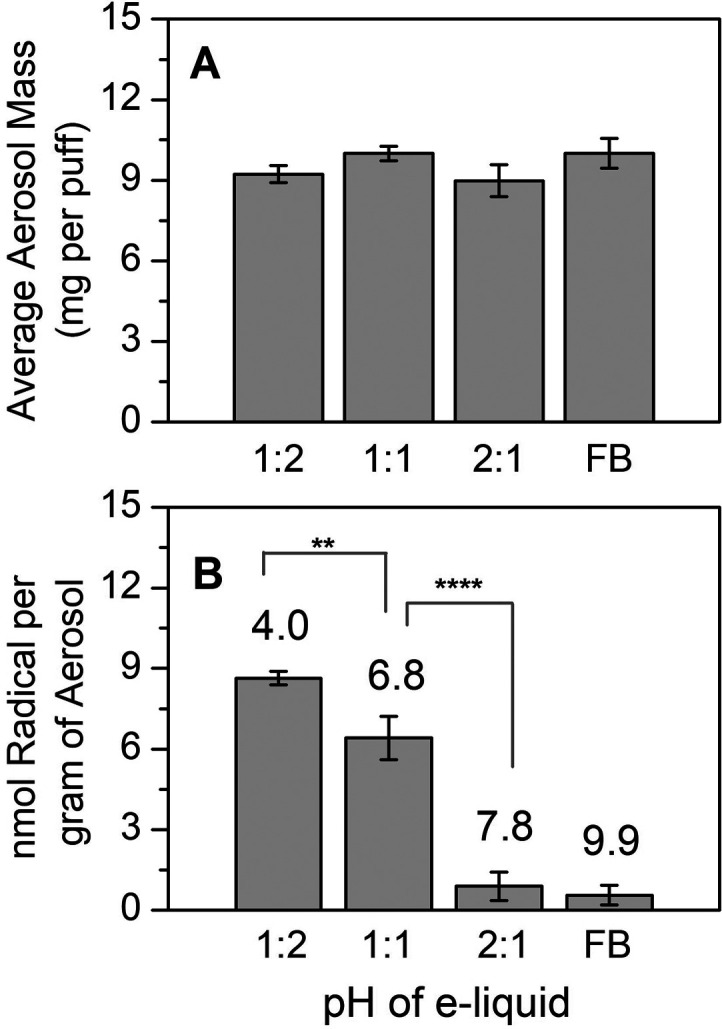
(A) Aerosol mass (mg per puff) and (B) radicals (nmol per gram of aerosol formed) from vaping nicotine benzoate salts with different nicotine to acid molar ratios compared to freebase (FB) nicotine. The corresponding measured pH are labelled above the column plot. Data were corrected for air background. Asterisks denote statistically significant differences using one-way ANOVA. **p < 0.05; ****p < 0.001. Triplicate data in A were compared against the mean ± SD, and triplicate data in B were compared using the denoted pairings.

Among the benzoate salts with different amounts of nicotine to acid ratios, the aerosol mass produced by vaping was relatively constant (not significantly different than the mean) and compared well to the aerosol mass formation from freebase nicotine (Table 3, Figure 7A). Interestingly, our results differ from the data from JUUL showing that BA nicotine salts delivered significantly more nicotine compared to freebase e-liquids.^44^ It is not clear whether the results are due to differences in e-liquid nicotine concentration, e-cigarette device design, temperatures tested, or other variables.^38^

**Table 3:** Aerosol Mass (mg per Puff) and Radicals (nmol and nmol per Gram of Aerosol Formed) (Mean ± SD) from Vaping Nicotine Benzoate Salts with Different Molar Ratios of Nicotine to Benzoic Acid

Nic:BA ratio	aerosol mass (mg/puff)	total radicals detected (nmol)	nmol radicals per g aerosol
1:2	9.23 ± 0.31	6.05 ± 0.40	8.64 ± 0.26
1:1	10.00 ± 0.27	4.48 ± 0.45	6.42 ± 0.80
2:1	8.99 ± 0.60	0.66 ± 0.38	0.90 ± 0.53
freebase	10.00 ± 0.55	0.36 ± 0.23	0.56 ± 0.37

**Figure 8:**
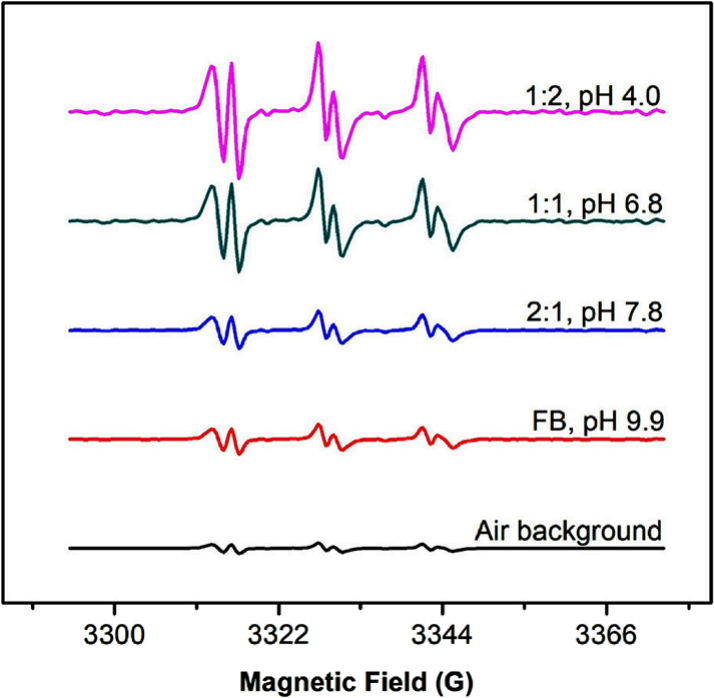
EPR spectra of PBN–radical adducts detected in the aerosol from vaping nicotine benzoate salts with different nicotine to benzoic acid molar ratios and corresponding pH, compared to freebase (FB) nicotine and air background. Data are normalized using the aerosol mass generated.

Even without acid present, the freebase solution formed a significantly higher radical yield than the air background (*p* < 0.05, Figure 8). In Section 3.2, we reported that BA nicotine salts yielded the highest concentrations of free radicals per gram of aerosol compared to the other organic acid salts tested. Here, Figure 7B and Figure 8 show that increasing BA concentration relative to a constant nicotine concentration is positively correlated to the radical production yield. Increasing concentrations of carboxylate ligands has been shown to increase the rate of oxidation of Fe(II) in Fenton reactions;^50^ thus, this effect could be due to a direct impact of the reagent concentration on the kinetics. Alternatively, increasing the organic acid concentration may increase the availability of redox-active free metals through increased leaching, which would also increase the concentration of the catalyst and accelerate the radical formation rate. Although a higher metal solubility at a higher solution acidity is well established, this has not yet been explicitly demonstrated in e-cigarettes. Finally, increasing the organic acid to nicotine ratio increases the distribution of the acid compared with the carboxylate form. Generally, carboxylates react faster with OH radicals compared to their acid form.^51^ Thus, a higher fraction of the acid form may decrease the radical scavenging ability of the nicotine salt solution. All three of these effects act in the same direction, and it is likely that the observation of higher ROS formation at a lower nicotine to acid ratio is due to a combination of factors.

## 4 Conclusions

It was found that the EPR spectra of the PBN-radical adducts of the vaped aerosol samples from BA nicotine salt in PG/VG match the PBN–OH spectra of the classical Fenton reaction of Fe(II) and hydrogen peroxide in water; thus, in consideration of other corroborating evidence,^33^ it may be concluded that ROS (and specifically OH radicals) drive the observations shown here. Of the eight organic acid conjugates in nicotine salts, BA, TA, and CA were the only acids for which radicals were detected in the aerosol above the air background. The highest radical yields were observed for BA and CA e-liquids, which was determined to result from a combination of radical production and scavenging processes. These spin-trapped ROS measurement data are consistent with recent *in vitro* results for vaping aerosols from BA and SLA nicotine salts from JUUL devices, wherein BA but not SLA salts were found to induce oxidative stress.^46^ Under the assumption that Fenton-like chemistry occurs in the vaping process, the results from CA and TA are expected, as these are well-studied ligands for Fenton-like reactions, but the observation that BA produced the highest radical yield is unexpected and novel. Given that BA and LA are the two most common organic acid additives in nicotine salt e-liquids in the market currently, the results here have implications for their safe usage in commercial e-cigarette vape solutions due to the negligible radical production for LA nicotine salts and the significant radical production from BA nicotine salts. Moreover, the aerosols from freebase nicotine had levels higher than the detected amounts for SLA, LA, LVA, SA, and MA nicotine salt e-liquids, which suggests that these acids can help scavenge free radicals and lower the concentration of radicals in the aerosol. The drastic differences in ROS formation between some of the acids tested in this work suggest that the structure and activity of organic acid additives (or their associated effects on solution acidity) are highly important for ROS formation in e-cigarette vapes.

The ROS yields at different acid/base ratios of BA nicotine salts increased with increasing BA to nicotine ratio. It is unclear if this observation is due to higher metal solubility, increasing organic acid ligand concentrations in the reactions of Fenton-like chemistry, altering the radical scavenging ability of organic acids, or other effects. As this work tested the acid to nicotine ratio effects only for BA nicotine salts, we are unable to evaluate whether the effect is generalizable in other e-liquids. It would be informative for future studies to quantify ROS for other common nicotine salts at other conditions. For example, while the aerosols from vaping LA nicotine salt at a 1:1 molar ratio (pH 7.3) were not found to yield significant ROS at the tested conditions, the ROS yields may be different at higher acid concentration. As e-liquid formulations on the market have a wide range of relative pH (and thus, organic acid to nicotine compositions) from pH < 4 to pH > 9,^11,52^ it is important for users, manufacturers and regulators to consider the implications of acid type and content on ROS formation in the inhaled aerosol and its related health outcomes.

OH radicals have been observed previously in a few studies involving e-cigarette aerosols,^33,53^ yet the origins of OH and other ROS in e-cigarette devices have remained an open question. It is often assumed that Fenton-like reactions drive ROS formation in e-cigarettes, although it is challenging to provide irrefutable evidence given the complexity of the chemical system tested here. This work cannot positively trace ROS formation back to Fenton-like chemistry, although the observation that known organic acid ligands for Fenton-like reactions produce higher radical yields in this study and the matching EPR spectra to that of OH provide corroboration that Fenton-like chemistry might be a contributing factor. However, the observation that vaping aerosol from the BA organic acid itself in JUUL devices did not induce oxidative stress outcomes in vitro, while the BA nicotine salt e-liquid did, challenges this idea. If Fenton-like chemistry drives ROS production in vapes, it is not clear why nicotine enhances the cellular oxidative stress response for BA-containing e-liquids, as nicotine is not understood to play any direct roles in Fenton chemistry. It must be emphasized that Fenton-like reactions are complex and that comparisons between the aqueous systems in which Fenton-like reactions are typically studied and e-liquids that have a low water fraction may not be straightforward. A number of different metals observed in the e-liquids and aerosol (e.g., Cu, Ag, Mn)^18^ can facilitate Fenton-like reactions^54^ along with numerous organic acid ligands, often with multidirectional behavior with respect to acidity, depending on the reactants.^55^ Furthermore, along with Fenton, there may be other reaction pathways that form ROS from the multitude of redox active trace metals present,^56,57^ heat from the coils, and catalytic surface area^58^ that may interact with the e-liquid additives. Thus, it is possible that the trends in the yields of OH and other ROS in e-cigarettes are not attributable to any one factor.

This research identified important new knowledge that provides guidance for the use, manufacture, and regulation of e-liquid formulations; however, limitations are also noted. First, as we followed the JUUL patent formulation, the nicotine concentrations used were on the lower end of the commercial range. Nicotine salt concentrations are available up to 5% commercially, more than double the amount used in this study, which can significantly alter the chemistry significantly. This work quantified the lower limit of ROS yields, as any concentration higher than the tested levels for BA solutions will most likely show higher levels of radicals. Also, the study of different organic acid conjugates was performed only at 1:1 molar ratio and pH 7 for consistency; it would be useful for future studies to investigate the unadjusted 1:1 nicotine salt of CA or with a 2:1 ratio, which produces closer to full neutralization. This may provide insight into how CA, TA, or other organic di- and triacid additives in nicotine salts can drive ROS formation. Furthermore, only single organic acid conjugates were tested here. Some commercial nicotine salts are made by combining multiple organic acids. For example, in the commercial mixture of BA and LVA,^59^ the addition of an organic acid that can scavenge free radicals may help reduce ROS levels from BA salts but further research is needed to support this hypothesis. It would also be instructive for future studies to investigate the effects of organic acid types and concentrations on the leaching of metals into the nicotine salt e-liquid as well as on the formation of other harmful constituents, such as carbonyls.
